# Adverse childhood experiences and risk of late-life dementia: a systematic review and meta-analysis

**DOI:** 10.1007/s00127-024-02676-4

**Published:** 2024-05-08

**Authors:** Moaz Elsayed Abouelmagd, Maickel AbdelMeseh, Amr Elrosasy, Hatem Abdelmoneim Eldeeb, Yehia Nabil

**Affiliations:** 1 Faculty of Medicine, Cairo University, Cairo, Egypt; 2 Faculty of Medicine, Alexandria University, Alexandria, Egypt; 3 Faculty of Medicine, Alazhar University, Cairo, Egypt; 4 Faculty of Medicine, Zagazig University, Zagazig, Egypt

**Keywords:** Dementia, Adverse childhood experiences, Cognitive impairment, ACEs, Abuse

## Abstract

**Background:**

Adverse childhood experiences (ACEs) refer to distressing events before age 18 that can lead to potential mental and physical health consequences. This systematic review and meta-analysis aimed to examine the association between ACEs and the risk of dementia in elderly adults who experienced ACEs during childhood, addressing the existing inconsistencies and methodological variations.

**Methods:**

A comprehensive search strategy was employed across key databases (PubMed, Web of Science, Scopus, and Embase) to identify relevant articles. Our primary outcome was ACEs-dementia risk, and our secondary outcome was mild cognitive impairment risk. A quality assessment was conducted using the Newcastle–Ottawa Quality Assessment Scale and GRADE. A random-effects model was utilized to calculate pooled odds ratios (ORs) and 95% confidence intervals (CIs). Subgroup analyses were performed to explore potential sources of heterogeneity and assess the reliability of the results.

**Results:**

Out of 1,376 screened papers, nine studies were included. The studies consisted of two case-control, one prospective cohort, and six retrospective cohort studies conducted in the UK, France, USA, China, and Spain. Five studies were of good methodological quality according to the NOS. according to the GRADE, all outcomes were classified as very low or low quality of evidence. A significant association was observed between ACEs and dementia risk (OR = 1.35; 95% CI 1.20, 1.52; *P* = 0.00001) and mild cognitive impairment risk (OR = 1.28; 95% CI 0.63, 2.62; *P* = 0.49). A meta-analysis by type of adversity revealed significant results for the maltreatment subgroup(OR = 1.30; 95% CI 0.07-1.58; *P* = 0.007; I² = 0%). Subgroup analysis based on the dementia definition revealed no between-subgroup difference (*P* = 0.71) between tool-based and register/criteria-based subgroups. No possibility of Publication bias was observed upon inspection of the funnel plot.

**Conclusion:**

Adverse childhood experiences may be associated with an increased risk of dementia. However, caution is warranted in interpreting these results due to the limited number of studies. Larger high-quality studies investigating the association between ACEs and dementia risk are needed to confirm the reliability of our results.

**Supplementary Information:**

The online version contains supplementary material available at 10.1007/s00127-024-02676-4.

## Introduction

Adverse childhood experiences (ACEs) encompass a range of stressful and traumatic events that have significant implications for the health of adolescents and adults. Globally, approximately one billion children aged between two and seventeen years are estimated to have experienced such violence at least once, with rates of 58% in North America and 42% in Europe [1, 2]. These experiences include various forms of abuse (physical and emotional), neglect (physical and emotional), household dysfunction (such as substance misuse, incarceration, chronic depression or mental illness, domestic violence, and disrupted family structure), and exposure to bullying and community violence [3].

Dementia, a neurodegenerative disease characterized by cognitive decline, represents a major global health concern. In 2015 alone, 47 million individuals were affected, with projections indicating that this number will triple by 2050 [4]. Dementia has been associated with structural and functional alterations in brain regions involved in cognition, including the hippocampus and prefrontal cortex, the same areas affected by childhood stress. These findings suggest that early life adversities may serve as risk factors for the development of dementia and cognitive impairment later in life [5, 6].

Stress and trauma have been linked to an increased risk of developing dementia. A recent meta-analysis (MA) revealed that the risk of dementia in individuals with posttraumatic stress disorder is 1.61–1.99 times greater than the risk in people without this disorder [7]. Childhood represents a crucial period in human development during which neurological connections are established. Any events occurring during this time can have long-lasting effects on health outcomes. A recent study has shown that ACEs are linked to an increased need for specialized healthcare, such as prescription medication, special therapies, and additional support services [8].

Although the exact connection between psychological trauma and dementia is not yet fully understood, proposed mechanisms suggest that neurobiological activity may be altered, stress-related pathways may be affected, cognitive stimulation may be reduced, and shared genetic vulnerabilities may play a role [7]. Furthermore, ACEs may impact dementia risk by influencing other associated risk factors. A recent study revealed that ACEs were positively correlated with increased dementia risk factors, including lower educational attainment, limited social contact, smoking, and clinical depression [9].

While previous systematic reviews have provided valuable qualitative insights into the relationship between ACEs and cognitive functions and Alzheimer’s disease in late life [10, 11], there is currently a lack of MA studies specifically examining the association between ACEs and the risk of developing dementia. Our systematic review and meta-analysis aims to fill this gap and provide a more comprehensive understanding of how ACEs impact the likelihood of developing dementia in late life.

## Methods

In this study, we followed the guidelines of the Preferred Reporting Items for Systematic Reviews and Meta-Analyses (PRISMA) and the Cochrane Handbook of Systematic Review and Meta-analysis. We registered the detailed protocol of this MA on open science framework (OSF) with the following DOI: 10.17605/OSF.IO/6EBDZ.

### Search strategy

A comprehensive search was conducted across major electronic databases (PubMed, Web of Science, Scopus, and Embase) for relevant keywords from conception through the 15th of December 2023.

For example, the PubMed search strategy used was as follows: ((childhood OR Child OR children OR “early life”) AND (Maltreatment OR adversity OR violence OR Trauma OR “Adverse childhood” OR neglect OR ACEs OR abuse OR stress) AND (Alzheimer OR dementia OR cognitive decline OR MCI) AND (observational OR COHORT OR CASE-CONTROL OR “case-control” OR RCT OR TRIAL OR register). Additionally, the references of the included studies were screened for potential articles to include.

### Study selection

To select the most relevant studies for this research, the titles and abstracts of the retrieved records from the search strategy were imported into Rayyan [12], an online platform for screening studies in systematic reviews. Duplicate records were eliminated, and two authors independently screened all abstracts. Conflicts were resolved by the first author. Subsequently, eligible studies underwent a full-text screening phase based on predefined inclusion and exclusion criteria.

#### Inclusion criteria

- English-language studies published in peer-reviewed journals.
- Participants experienced adverse childhood experiences (ACEs) before the age of 18, including violence, abuse, neglect, witnessing violence in the home, family member’s death, living in an unstable house with substance use/mentally ill/incarcerated parents, traumatic experiences, etc.
- Participants aged > 60 at follow-up.
- Dementia or cognitive impairment was defined using validated diagnostic criteria, tools, or medical records.
- The studies included sufficient data, such as odds ratios (ORs), risk ratios (RRs), hazard ratios (HRs), or data for calculation.
- Longitudinal study design (case-control or cohort).
- A healthy control group was included.

#### Exclusion criteria

- Studies that focused on head trauma, childhood diseases, or physical injury that may lead to dementia or mild cognitive impairment (MCI).
- Studies on high-risk medical populations (e.g., diabetes, hypertension).
- The full texts of the studies were unavailable after contacting the corresponding author.

### Data extraction

Two authors extracted relevant data from the included studies into a data extraction Google Sheet. The extracted data for each study included the study ID, study design, country, baseline characteristics, sociodemographic characteristics, measurement tools for cognition and early-life adversity, number of participants, length of follow-up, adjustment factors, outcomes, and key findings.

### Methodological quality assessment

We used the Newcastle–Ottawa Quality Assessment Scale (NOS) to assess the quality of the studies. The NOS consists of eight questions. This scale evaluates three parameters of quality (selection, comparability, and outcome) and includes specific items that vary slightly for case-control and cohort studies. Each item on the scale can receive a maximum score of 1 point, except for comparability, which can be adjusted to the specific topic of interest, and a maximum score of 2 points, with the highest overall score being 9 [13]. To convert the NOS to fit the standards set by the Agency for Healthcare Research and Quality—good, fair, and poor—the following thresholds are applied [14]:

Good quality: 3 or 4 stars in the selection domain 1 or 2 stars in the comparability domain AND 2 or 3 stars in the outcome/exposure domain.

Fair quality: 2 stars in the selection domain 1 or 2 stars in the comparability domain AND 2 or 3 stars in the outcome/exposure domain.

Poor quality: 0 or 1 star in the selection domain; 0 stars in the comparability domain; and 0 or 1 star in the outcome/exposure domain.

Additionally, the GRADE approach was applied to rate the overall quality of evidence and strength of recommendations for each outcome of interest. This approach considers factors such as risk of bias, inconsistency, indirectness, imprecision, and publication bias. The confidence in the estimates is categorized as high, moderate, low, or very low [15].

### Statistical analysis

The primary outcome of interest was the odds of dementia in ACEs-exposed elderly individuals with 2 or more ACEs, and the secondary outcome was the odds of MCI in ACEs-exposed elderly individuals. The effect sizes of the included studies were expressed as odds ratios (ORs), risk ratios (RRs), or hazard ratios (HRs). If multiple outcomes were reported, the outcomes with the best diagnostic criteria, highest number of cases, and best adjustment were chosen. An OR of 1 indicates no association, an OR between 0 and 1 indicates a negative relationship and an OR greater than 1 indicates a positive relationship, indicating that early-life adversity is associated with a greater risk of dementia/MCI.

Heterogeneity between studies was assessed visually using forest plots and the I² test. I² values less than 50% indicated insignificant heterogeneity, while values ≥ 50% indicated substantial heterogeneity. A random-effects model (DerSimonian‒Laird) was used to calculate the pooled OR and 95% confidence interval (CI) to account for within- and between-study heterogeneity originating from different effect sizes and/or ACE measurement tools. Sensitivity and subgroup analyses were performed to investigate potential sources of heterogeneity and to test the stability of the results. Subgroup analyses were also conducted for primary outcomes based on the type of adversity, definition of dementia, and quality of evidence if there were at least 2 studies for each subgroup.

### Publication Bias

Funnel plots of the primary MA model were visually examined to assess potential publication bias. The uneven distribution of studies around the pooled effect estimate suggested the possibility of publication bias. Additionally, Egger’s test was used.

## Results

### Search results and characteristics of the included studies

**Fig. 1:**
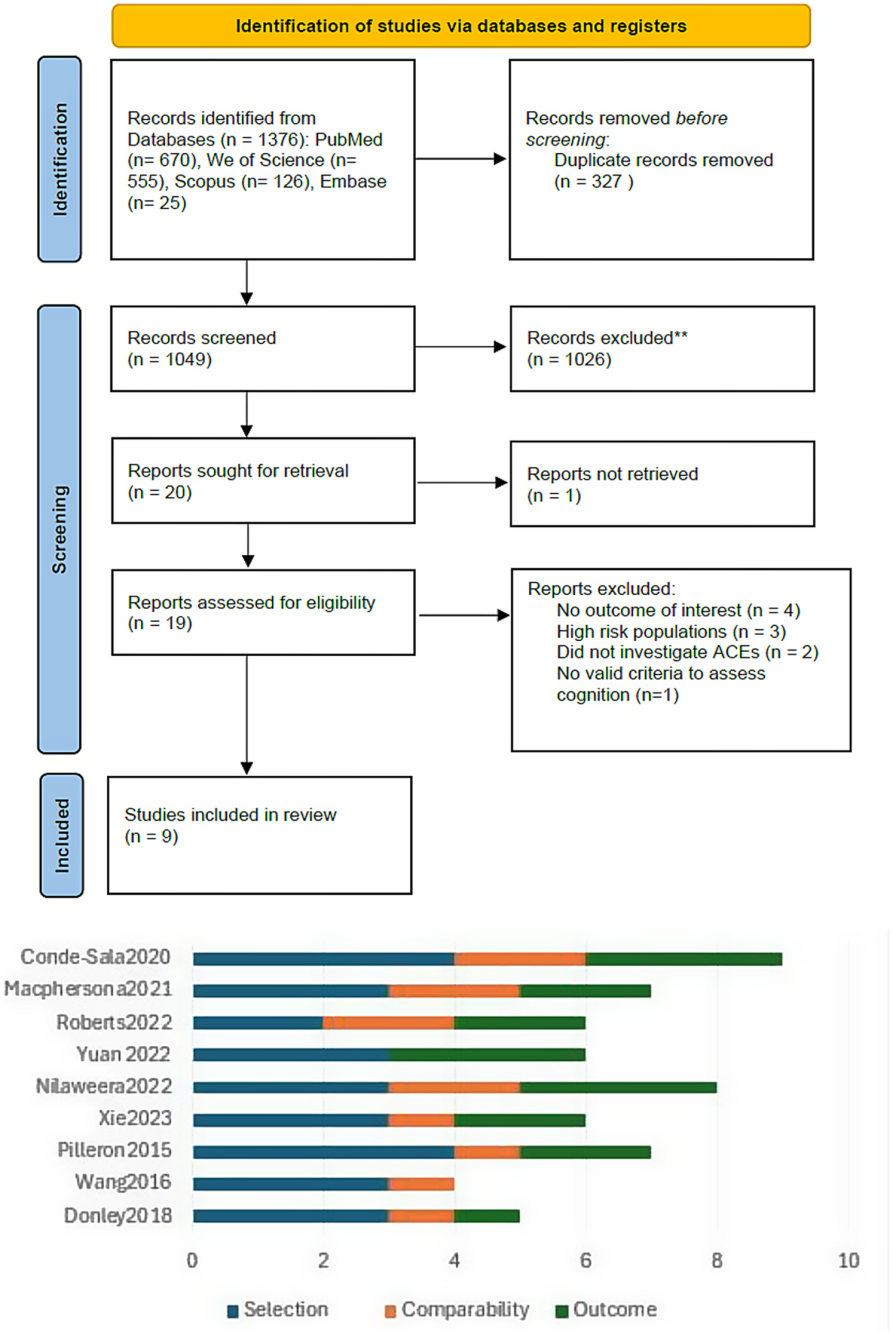
upward is the PRISMA flow diagram showing the details of the study selection process. Downward is the quality assessment summary of the included studies according to the Newcastle-Ottawa Quality Assessment Scale

We retrieved 1376 papers through a literature search; 327 of these were duplicates, and we ultimately included nine studies [16–24] that presented data from 283,108 participants in the MA (see Fig. 1; Table 1). We included two case-control studies [21, 23], one prospective cohort study [19], and six retrospective cohort studies [16–18, 20, 22, 24]. The studies included data from various countries, such as the UK, France, the USA, China, and Spain. The detailed characteristics of the included studies are shown in (Table 1.**)**

**Table 1:** Summary of included studies

Study ID	Study Design	Country	Population (No. of events)	Definition of dementia/ MCI	Measurement of adversity	Variables adjusted	Effect size	Quality assessment (Newcastle Ottawa Scale)
Xie 2023 [16]	Retrospective cohort	UK	1,45,558 (497)	ICD-9 and ICD-10	Three questions Childhood screener	1,2,3,4,5,11,12,13,24	Traumatic events > 3 were significantly associated with dementia (HR = 1.62, 95% CI: 1.07–2.22)	Good quality
Nilaweera 2022 [17]	Retrospective cohort	France	1549 (154)	MMSE and DSM-IV criteria	25-item questionnaire (modified version of the Childhood Trauma Questionnaire)	1,2,3,8,23	ACE 3–4 was not significantly associated with dementia (OR = 0.9, 95% CI: 0.62–1.31)	Good quality
Yuan 2022 [18]	Retrospective cohort	China	7222 (1033)	MMSE	A survey including 11 types of adversity based on Adverse Childhood Experiences International Questionnaire	1,2,5,21,22	ACE = 3 was significantly associated with dementia (OR = 1.37, 95% CI: 1.12–1.68)	Poor quality
Roberts 2022 [19]	Prospective cohort	USA	1755 (752)	AD8-Washington University Dementia Screening Test	Adverse Childhood Experiences Questionnaire.	1,14,20	ACE > = 4 associated was significantly associated with dementia (RR = 1.48 95% CI: 1.22–2.47)	Fair quality
Macpherson 2021 [20]	Retrospective cohort	UK	56,082 (52)	AD8: The Washington University Dementia Screening Test	Childhood Trauma Screener (CTS)	1,2,19	Childhood maltreatment class was significantly Associated with dementia (HR = 1.32, 95% CI: 1.02–1.71)	Good quality
Conde-Sala 2020 [21]	Case-control	Spain	65,997	Self-reported proxy-reported diagnosis of dementia by a doctor	Year of parental death and year of birth of participants	1,2,3,5	The death of a parent was significantly Associated with dementia (OR = 1.54, 95% CI: 1.35–1.76)	Good quality
Donley 2018 [22]	Retrospective cohort	USA	2682 (360)	National health registers	childhood stress index questionnaire	1,3,5,9,11,13,16,17,18	childhood stress index was significantly associated with dementia (HR = 1.86, 95% CI: 1.01–3.39)	Poor quality
Wang 2016 [23]	Case-control	China	137 (76)	MoCA and MMSE	Childhood Trauma Questionnaire - Brief Version (CTQ-RF) Adulthood Life Events Questionnaire	1,6,7,13,14	ACE 3–4 was not significantly Associated with MCI (HR = 0.72, 95% CI: 0.25–2.08)	Poor quality
Pilleron 2015 [24]	Retrospective cohort	France	1,772 (135)	CSI-D	Persson and Skoog questionnaire	1,2,3,4,8,9,10,11	Total early traumatic events was not significantly associated with dementia OR = 1.22, 95% CI: 0.86–1.72	Good quality

No. number; HR: hazard ration; OR: odds ratio; RR: risk ratio; CI: confidence interval; ACEs: Adverse Childhood experiences

MCI: Mild Cognitive Impairment; SLEs: Stressful Life experiences; ICD: International Classification of Diseases; MMSE: The Mini-Mental State Examination; MoCA: Montreal Cognitive Assessment ;

(1) Age (2) Sex (3) Education (4) Residency (5) Socioeconomic status (6) Emotional abuse (7) Physical abuse (8) Depressive Symptoms (9) BMI (10) Alcohol consumption 11. Smoking 12. Stroke 13. Diabetes 14. Adult adverse events 15. Parenteral separation 16. Heart disease 17. Blood pressure 18. Cholesterol 19. Ethnicity 20. Race 21. Adulthood health behaviors 22. Adulthood health status factors 23. ApoE 24. Medical History

**Table 2:** Summary of analyses and Quality of outcomes according to GRADE

Outcomes	Number of studies	Pooled OR (95% CI)	Heterogeneity (I2)	Test of subgroup difference (P value)	evidence (GRADE)
Primary Analysis				0.64	
Dementia	7	1.35 (1.20- 1.52)	16%		Very Low^a^
MCI	2	1.28 (0.63- 2.262)	51%		Very Low^a^
Type of ACE				0.06	
Maltreatment	3	1.30 (1.07- 1.58)	0%		Low
Death of parent	3	1.15 (0.76- 1.73)	84%		Very Low^b^
Dementia definition				0.0001	
Tool	4	1.41 (1.25- 1.60)	0%		Low
Register/ Diagnostic criteria	3	1.30 (1.0.86 1.97)	42%		Low
Quality of studies				0.64	
Good or fair	5	1.31 (1.11- 1.53)	33%		-
Low	2	1.35 (1.17- 1.71)	0%		-

**OR: odds ratio; MCI: mild cognitive impairment; CI: Confidence interval**

**Explanations**

a. Downgraded for indirectness as different Adversity measurement tools were included

b. Downgraded for inconsistency due to high heterogeneity

**GRADE Working Group grades of evidence**

**High certainty**: we are very confident that the true effect lies close to that of the estimate of the effect

**Moderate certainty**: we are moderately confident in the effect estimate: the true effect is likely to be close to the estimate of the effect, but there is a possibility that it is substantially different

**Low certainty**: our confidence in the effect estimate is limited: the true effect may be substantially different from the estimate of the effect

**Very low certainty**: we have very little confidence in the effect estimate: the true effect is likely to be substantially different from the estimate of effect

### Quality assessment of the included studies

Among the cohort and case-control studies, six were classified as having good or fair methodological quality [16, 17, 19–21, 24] (see Fig. 1; Table 1). These studies exhibited strong adherence to methodological principles and minimal risk of bias, while the remaining studies were categorized as poor quality [18, 22, 23]. According to the GRADE system, all the included outcomes yielded very low-quality evidence. The details and explanations are clarified in Table 2.

### Risk of dementia and MCI

**Fig. 2:**
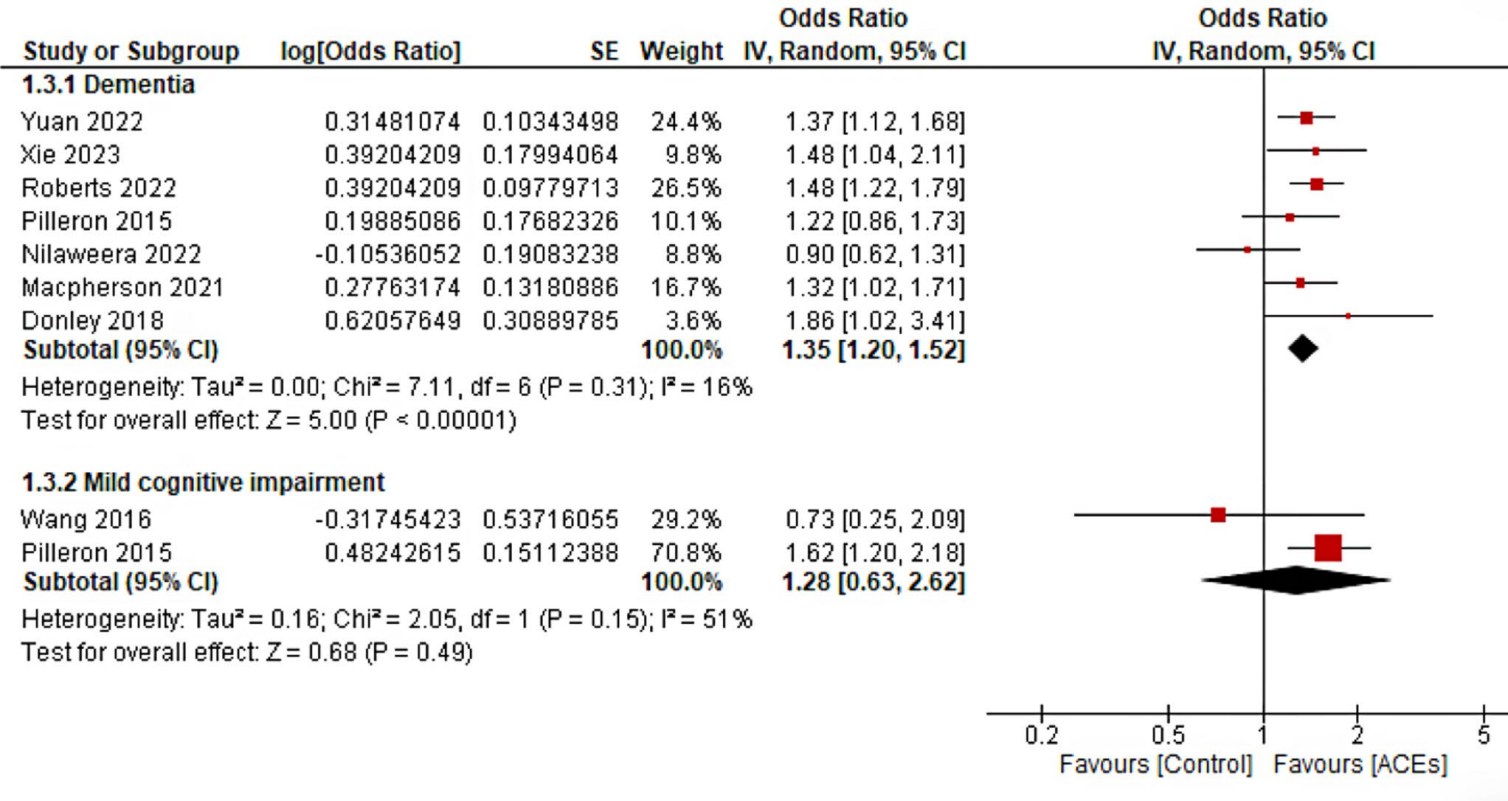
Forrest plot of the association between ACEs and risk of Dementia and Mild cognitive impairment

Seven studies were included in the dementia risk analysis [16–20, 22, 24], and 2 studies were included in the MCI analysis [23, 24]. All the studies examined ACEs as a cumulative score of 2 or more ACEs through questionnaires containing several domains. However, one study was excluded from this analysis because it measured only 1 adverse event—parents’ death—to avoid heterogeneity [21]. The definition of dementia or MCI was based on screening tools [diagnosis by a neurologist or registers using International Classification-of-diseases Version 8 (ICD-8)]. The results showed that exposure to ACEs was associated with dementia risk (OR = 1.38; 95% CI 1.25–1.52); *P* < 0.00001), with no significant heterogeneity (I2 = 0%). However, there was no significant difference in the risk of developing MCI (OR = 1.28; 95% CI 0.63–2.62); *P* = 0.49), and there was moderate heterogeneity (I² = 51%, *P* = 0.15) (Fig. 2**)**.

#### Publication bias

**Fig. 3:**
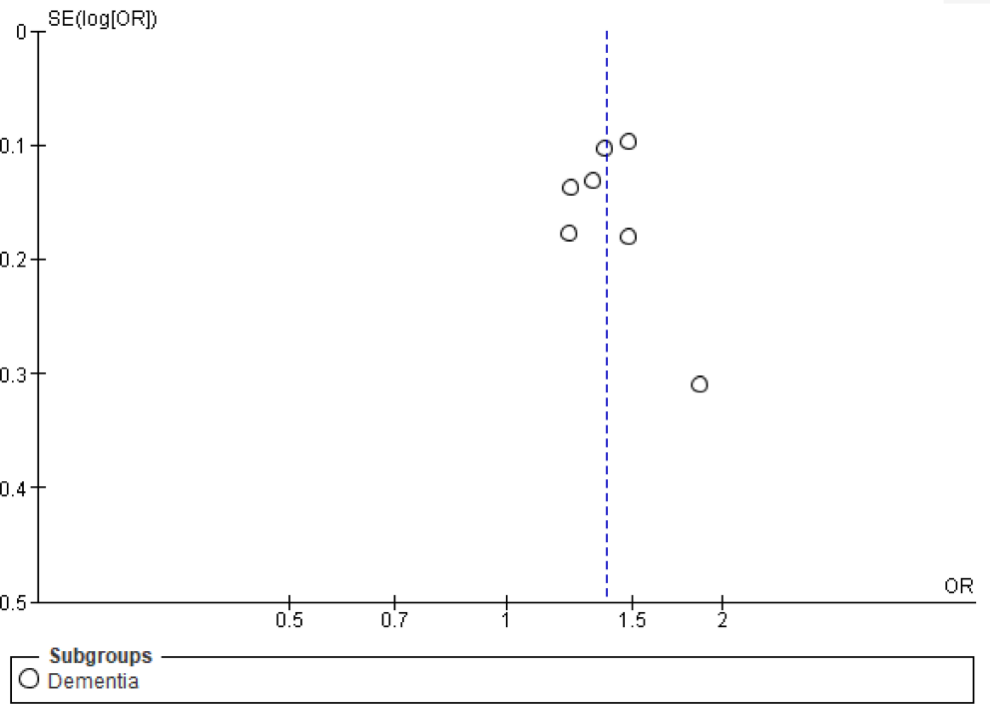
**Publication** bias of the association between ACEs and dementia risk

Inspection of the funnel plot of dementia risk outcome didn’t reveal significant asymmetry and the results of Egger’s test were not significant (*P* = 0.473). (Fig. 3)

### Risk of dementia based on type of adversity

Six studies reported specific types of childhood adversity, especially childhood maltreatment and the death of a parent. Childhood maltreatment showed a slight significant association with the risk of dementia (OR = 1.30; 95% CI 0.07-1.58; *P* = 0.007; I² = 0%), while the death of a parent showed no significant association (OR = 1.15; 95% CI 0.76–1.73; *P* = 0.002; I² = 84%). The test of subgroup differences was not significant (0.58). Heterogeneity was resolved in the second subgroup after excluding Conde-Sala 2020 in the second subgroup [23] (supplementary material Fig. 1, Fig. 2).

### Risk of dementia based on the definition of dementia

Seven studies were included in the subgroup analysis based on the definition of dementia. Three studies defined dementia through registries and diagnostic criteria, while 4 studies defined dementia based on cutoff points of screening tools. The tool-based subgroup exhibited a significant association between ACEs and dementia (OR = 1.41; 95% CI 1.25–1.60; P < 0.00001; I² = 0%), but the register-based subgroup did not exhibit a significant difference (OR = 1.30; 95% CI 0.86–1.97; P = 0.49; I² = 42%). No significant heterogeneity was found in either subgroup analysis, and the test for subgroup differences was not significant (P = 0.71). (supplementary material Fig. 3)

### Risk of dementia based on the quality of the included studies

Seven studies were included in the subgroup analysis based on the quality of the studies: 5 were of good or fair quality, and 2 were of low quality. The associations between ACEs and the risk of dementia were significant in both the good/fair subgroup (OR = 1.31; 95% CI 1.11–1.53; P < 0.0010; I² = 33%) and the low subgroup (OR = 1.41; 95% CI 1.17–1.71; P = 0.0004; I² = 0%). The test of subgroup differences was not significant (P = 0.74), and no significant heterogeneity was found within the subgroups (supplementary material Fig. 4).

## Discussion

Recent research predicts that the prevention of ACEs is associated with depression, stroke, coronary heart disease, diabetes, and obesity [25, 26]. Our MA aimed to investigate the association between ACEs and the risk of developing dementia and MCI in adulthood. We found a significant relationship between exposure to ACEs and an increased risk of dementia. Subgroup analyses were also conducted to explore specific adverse events, diagnostic criteria, and study quality.

In terms of specific childhood adversities, our findings showed that childhood maltreatment was slightly significantly associated with increased dementia risk, while the death of a parent was not. However, these results are limited by the low number of included studies in each of these analyses and should be interpreted with caution. Regarding diagnostic criteria, individuals diagnosed based on tools showed a significant association, while those diagnosed based on registers did not. When considering study quality, both acceptable and low-quality studies demonstrated a significant association with the risk of dementia. Although most of the studies in our analysis adjusted their results for more than 3 factors, there may be other confounding factors that drive the relationship between traumatic experiences and dementia, which were not adequately accounted for in the analyzed studies.

Three studies [16, 17, 19] in our primary analysis of dementia reported outcomes as cumulative ACEs score > 3 experiences. While three of them reported significant results, Nilaweera et al. [17]. did not. This study was of good quality and used the largest questionnaire of 25 items for adversity. The absence of findings is mostly due to selection bias, as authors included only participants older than 65 years at baseline who were followed up for 14 years. This may have induced selection bias, as participants who had dementia were excluded during the selection stage.

Pilleron et al. [24]. also did not find significant results for dementia but did find significant results for MCI. This may be attributed to the limited number and types of adversities asked about in their questionnaire. This questionnaire included five life events before the age of 16 (death of a parent, divorce of parents, growing up with one parent, different guardians, and extreme poverty); these five life events are substantially different from the other measurement tools used in our primary MA, where most studies included questions about emotional, physical and/or sexual abuse.

Yuan *et al*. reported an independent relationship between ‘Child abuse’ and a higher risk of cognitive impairment in elderly Chinese people, which our pooled analysis confirmed. Conde-Sala *et al*. reported an increased risk of dementia associated with early parental death. However, our MA did not find a significant relationship between maltreatment or parental death and an increasing risk of dementia.

Various methods were employed to measure ACEs in the studies included in our analysis. Some studies utilized structured questionnaires to assess cumulative ACEs, while others relied on self-reports focusing on specific ACEs. Several studies have used validated questionnaires to assess several ACEs. One study [20] used the Childhood Trauma Screener (CTS) [27], two studies [17, 23] used modified versions of the Childhood Trauma Questionnaire (CTQ) [28, 29], and two studies [18, 19] used the Adverse Childhood Experiences International Questionnaire (ACE-IQ) [30] or a modified version of it. However, some studies have used simple questions to assess several ACEs [16, 22, 24] or a record to define the death of parents in childhood [21].

Cumulative scores ACE methods such as ACE-IQ and CTQ, involve summing the number of ACEs reported by each individual. Although these cumulative scores do not capture the severity of the experiences, they are widely accepted and favored in ACE research due to their greater predictive power compared to single-item scores [31, 32]. The ACE-IQ is currently the standard tool for measuring ACEs in clinical research [30]. Accordingly, our primary analysis included studies using cumulative or composite scores, and a secondary analysis was conducted based on specific ACEs, which were reported to be significant only for child maltreatment. It is worth noting that the number and categories of ACE items varied across studies, which may be accounted for by the ACEs dose-response effect [33, 34].

The majority of the evidence in this study comes from retrospective studies, which should be considered when interpreting the results. Retrospective studies have reported discrepancies when compared to prospective studies on ACEs [35]. In our included studies, ACE data were mostly obtained through subjective measures, such as self-reports. It is crucial to acknowledge that subjective measures of ACEs may introduce recall bias, as individuals may have difficulties remembering or may choose not to report past ACEs. Previous research has indicated a moderate correlation between subjective measures and objective measures, such as records or registries [36]. Nonetheless, this highlights the significance of the subjective nature of each child’s experience with traumatic events and its potential long-term effects.

Previous studies highlighted the possible association between ACEs and late-life cognition. Corney et al. [10]. suggested a link between ACEs and Alzheimer’s disease, although their study included only two studies and focused solely on Alzheimer’s disease. Patel et al. [11] conducted a systematic review of cross-sectional and cohort studies on the association between ACEs and late-life cognitive impairment. While their qualitative synthesis provided valuable insights, they did not explore the association between ACEs and dementia risk. In this study, we aimed to build upon previous research by conducting a quantitative synthesis with a rigorous methodology, focusing on the association between ACEs and dementia risk. Our analysis included cohort and case-control studies, incorporating six studies that were not included in the latest systematic review [11].

### Strengths and limitations

Our study represents the first systematic review and MA examining the association between ACEs and the risk of late-life dementia. We adhered to the PRISMA guidelines for systematic reviews, ensuring a comprehensive and rigorous approach. The strengths of our study include the use of a robust methodology: we implemented strict inclusion and exclusion criteria to focus specifically on ACEs and dementia risk. We utilized the adjusted Newcastle–Ottawa Scale and GRADE criteria to assess the quality of the evidence. However, it is important to acknowledge the potential for human bias at any stage of the study.

This MA has certain limitations that should be acknowledged. First, the retrospective observational study designs utilized in the included studies did not establish causal relationships but rather indicated associations. However, given the nature of our study, this design was the most practical and suitable approach. Second, the number of included studies and the number of events may limit the generalizability of our results. Third, the incorporation of different measurement tools for ACEs and dementia may have introduced heterogeneity. However, we addressed this by employing a random-effects model in our MA and specifically included studies that utilized composite scores for ACEs in our primary analysis. Additionally, we conducted a subgroup analysis to explore different potential sources of heterogeneity. Fourth, the quality of evidence assessed using the GRADE approach was determined to be very low for all outcomes, underscoring the importance of additional high-quality studies to strengthen the overall evidence base. Fifth, the grey literature was not thoroughly explored which may increase the possibility of publication bias. Last, it is worth noting that our study included only publications in English, which may have resulted in the exclusion of relevant studies published in other languages.

### Recommendations for future studies

Future studies should consider (1) conducting larger high-quality controlled case‒controls or retrospective cohort studies; (2) using validated cumulative ACEs score measurement tools such as the Adverse Childhood Experiences International Questionnaire and reporting data on specific adversities whenever applicable; (3) incorporating both subjective and objective ACE measurements to overcome reporting bias; (4) preferring the use of diagnostic criteria for dementia over screening tools for identifying dementia cases; and (5) including diverse representative populations from different races, education, and socioeconomic statuses.

## Conclusion

In conclusion, the findings of this systematic review and meta-analysis suggest that ACEs may be associated with an increased risk of dementia in elderly adults. However, it is important to exercise caution when interpreting these results due to the limited number of studies and the very low quality of evidence provided by the grading system. These findings underscore the importance of recognizing early-life adversities in assessing dementia risk. However, additional high-quality longitudinal studies are needed to support our findings. ACEs have already been linked to many negative health and developmental outcomes. Understanding the association between ACEs and dementia risk has crucial clinical implications. Healthcare professionals should be attentive to children with a history of ACEs and should implement early interventions and support systems to mitigate the long-term consequences for cognitive health. Tailored strategies for individuals with specific adversities or diagnosed through certain criteria may enhance preventive measures.

To our knowledge, this is the first attempt to quantify and analyze studies on the association between ACEs and dementia risk.

## Electronic supplementary material

Below is the link to the electronic supplementary material.

Supplementary Material 1

## Data Availability

The data that support the findings of this study are available from the corresponding author upon reasonable request. Guarantor: Moaz Elsayed

## Abbreviations

ACEs: Adverse Childhood experiences
MCI: Mild Cognitive Impairment
OR: odd ratio
RR: risk ratio
HR: Hazard ratio
NOS: Newcastle-Ottawa Quality Assessment Scale
CI: confidence interval
MA: Meta-Analysis
SLEs: Stressful Life experiences
ICD: International Classification of Diseases
MMSE: The Mini-Mental State Examination
MoCA: Montreal Cognitive Assessment
CSI-D: Community Screening Interview for Dementia
