# Supplementary material for: Adverse childhood experiences and risk of late-life dementia: a systematic review and meta-analysis
Source: Soc Psychiatry Psychiatr Epidemiol. 2024 May 8;60(5):1087–98. doi: 10.1007/s00127-024-02676-4 (PMC12119739; doi:10.1007/s00127-024-02676-4)
Supplement: Supplementary file 1 — Supplementary Material 1 [file 127_2024_2676_MOESM1_ESM.docx]

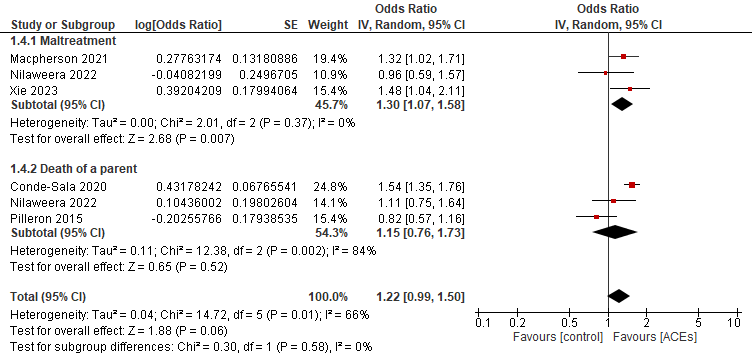
Fig.1 Forrest plot of Risk of dementia based on the type of adversity


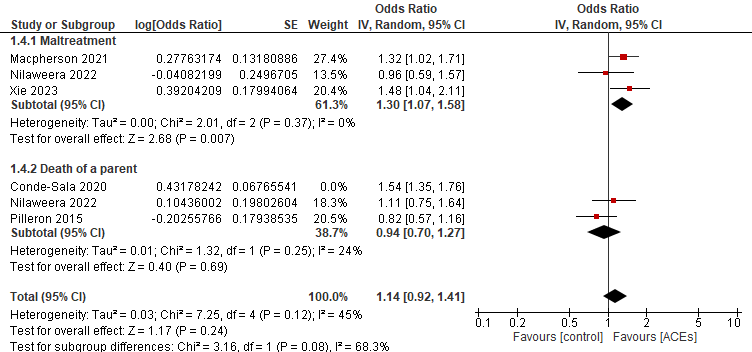


Fig.2 Forrest plot of sensitivity analysis of Risk of dementia based on the type of adversity


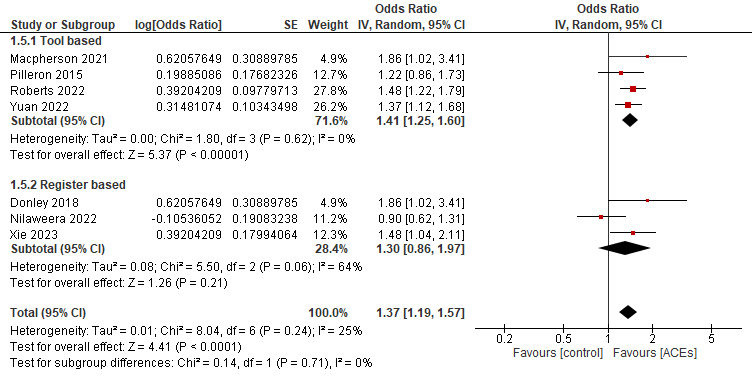


Fig.3 Forrest plot analysis of Risk of dementia based on the Definition of dementia


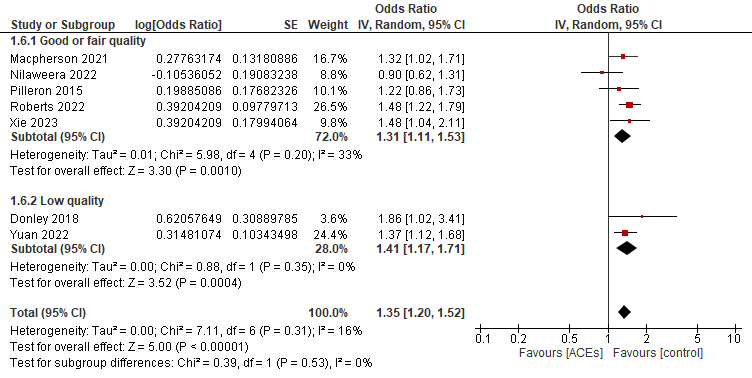


Fig.4 Forrest plot of Risk of dementia based on Quality of studies
